# Adjunctive Use of Active Compounds such as Chlorhexidine in the Nonsurgical Treatment of Peri-Implant Mucositis for Oral Health: A Systematic Review and Meta-Analysis

**DOI:** 10.1155/2022/2312784

**Published:** 2022-08-27

**Authors:** Rui Zhao, Sixin Liu, Yiming Liu, Shuxia Cui

**Affiliations:** ^1^Department of Stomatology, The First Affiliated Hospital of Zhengzhou University, Zhengzhou, 450052 Henan, China; ^2^University of Michigan School of Dentistry, 1011 N University Ave, Ann Arbor, MI 48109, USA

## Abstract

**Background:**

Peri-implant mucositis (PiM) is characterized as a reversible inflammatory change of the peri-implant soft tissues without alveolar bone loss or continuing marginal bone loss. Without proper control of PiM, the reversible inflammation may advance to peri-implantitis (PI). Mechanical debridement (MD) by the implant surface is the most common and conventional nonsurgical approach to treat PiM but with limitations in complete resolution of diseases. For more than a decade, chlorhexidine (CHX) and active compounds has been investigated in the treatment of PiM. Therefore, the aim of this systematic review and meta-analysis was to evaluate the efficacy of CHX treatment in combination with MD versus MD alone or MD+placebo in patients with PiM on their oral health problems.

**Methods:**

A search using electronic databases (Ovid MEDLINE, EMBASE, Science Direct databases, and Cochrane Central Register of Controlled Trials) and a manual search up to May 2022 were performed independently by 2 reviewers and included eligible randomized controlled trials (RCTs) comparing MD+CHX versus MD alone or MD+placebo. The assessment of quality for all the selected RCTs was conducted according to the Cochrane Handbook for Systematic Reviews of Interventions. Disease resolution of PiM (absence of BOP), IPPD reduction, IBOP% reduction, and PI% reduction after treatment as primary outcomes were selected as the primary outcomes. Weighted mean differences (WMD) and 95% confidence interval (CI) were for continuous outcomes, and odds ratio (OR) and 95% CI was for dichotomous outcomes using random effect models. This review is registered on the PROSPERO database (CRD42020221989).

**Results:**

After independent screening, nine eligible studies were included in this systematic review and meta-analysis. Meta-analysis showed OR of disease resolution between test and control groups amounted to 1.41 (95% CI (0.43, 4.65), *P* = 0.57, *I*^2^ = 65%) not favoring adjunctive CHX treatment over MD alone. Through subgroup analysis, the results indicated that oral irrigation of CHX may have more benefits on the resolution of PiM. Similarly, CHX did not significantly improve IPPD reduction at both short-, medium-, and long-term follow-up. Only a short-term effect has been observed at IBOP% reduction (WMD = 13.88, 95% CI (10.94, 16.81), *P* < 0.00001, *I*^2^ = 9%), IPI reduction (WMD = 0.12, 95% CI (0.09, 0.14), *P* < 0.00001, *I*^2^ = 0%), and FMPPD reduction (WMD = 0.19 mm, 95% CI (0.03, 0.35), *P* = 0.02, *I*^2^ = 0%) with adjunctive CHX application.

**Conclusion:**

Adjunctive CHX application may have some benefits to improve the efficacy of MD in PiM treatment by reducing IBOP%, IPI, and FMPPD in short-term. But these benefits disappeared at medium- and long-term follow-up. In order to achieve better disease resolution of PiM, adjunctive CHX irrigation with MD may be suggested and has positive potential. Well-designed large clinical trials are needed in future.

## 1. Introduction

Long-term success of dental implants supported fixed prostheses depends on healthy situation of soft and hard tissues surrounding implants [1]. Many complications such as biological complication including PiM and PI may affect the tissues surrounding implants and cause dental implant failure. PiM is characterized as a reversible inflammatory change of the peri-implant soft tissues without alveolar bone loss or continuing marginal bone loss, while peri-implatitis (PI) is a chronic inflammation result in progressive loss of supporting bone [2, 3]. According to recent meta-analysis and systematic review, PiM occurred in approximately 21%–88% of subjects with implants and 9%–51% of the inserted implant sites, and weighted mean prevalence was 46.8% and 29.5%, respectively [4]. Moreover, without proper control of PiM, the reversible inflammation may advance to PI causing irreversible bone loss which is still a challenging complication because of the absence of predictable, evidence-based protocol [5]. Therefore, the management of PiM has critical clinical significance. It has been shown that the absence of signs of clinical inflammation is necessary for concluding healthy condition of peri-implant [6].

The basic treatment of PiM is to eliminate or suppress of bacterial biofilm and periodontal pathogens [7]. The mechanical debridement (MD) by implant surface using curettes is the most common and conventional nonsurgical approach [2]. However, limitations of this protocol still exist in the complete resolution of PiM due to the complex abutment connection geometry and the implant neck morphology [8]. Besides MD, many adjunctive therapies have been applied to increase the inflammation control and antimicrobial effect, such as air polishing, photodynamic treatment, local use of systemic antibiotics, and probiotics [9–12]. Among all peri-implant therapy adjuvants, chlorhexidine (CHX), a broad-spectrum bacteriostatic and bactericidal agent has been commonly used since 1950s and proved in dental plaque control and prevention of bacterial biofilm [13–16]. The use of CHX in dentistry and oral healthcare continues to be widespread and common usage includes (but is not limited to): the management of oral hygiene, dental plaque, and caries; gingivitis, periodontitis, and peri-implant disease; root canal therapy, oral surgery, and associated complications; oral mucosal disease and as a prerinse to reduce aerosolisation of microbes during dental procedures [14]. For example, CHX as a mouthwash applied in dentistry, not only have antimicrobial effect on local part but also have full-mouth effect on bacteria, fungus, and virus causative for various of different oral infectious disease [17]. For more than a decade, several studies have been conducted to investigate the adjunctive effectiveness of CHX in the nonsurgical treatment of PiM but with inconclusive results[13, 18–24]. Three factors may be explained to the heterogeneity in outcomes: (1) different case definitions of PiM in clinical studies; (2) the wide range of CHX concentration, frequency, and treatment duration applied in studies; and (3) different delivery systems of CHX, such as mouthwash, spray, and gel formulations. To date, only one systematic review included 4 studies to 2016 with weak quality of evidence suggested that the adjunctive CHX therapy may not improve outcomes with nonsurgical management of PiM [25].

Therefore, the purpose of this systematic review was to analyze the available scientific literature and conduct meta-analysis to evaluate whether adjunctive CHX therapy is effective in improving outcomes when compared with MD alone or combination with placebo in the nonsurgical treatment of PiM in humans.

## 2. Methods

### 2.1. Protocol

This systematic review was registered in PROSPERO (CRD42020221989) and conducted in accordance with the guidelines of the PRISMA statement (Preferred Reporting Items for Systematic Reviews and the principles of PRISMA statement) [26] and Cochrane Handbook for Systematic Reviews of Intervention [27].

### 2.2. Focused Question

The focused question of this systematic review was addressed according to the population, intervention, comparison, outcome (PICO), and principle [28]: “What is the effect of adjunctive CHX therapy in patients undergoing nonsurgical treatment of PiM when compared with MD alone or MD + placebo?”

Population: patients diagnosed with PiM based on similar case definitions in the publications.

Intervention: the use of CHX as adjuncts in nonsurgical treatment.

Comparison: the nonsurgical treatment without the use of CHX or combined with placebo.

Outcomes: the changes of signs of peri-implant mucosal inflammation, such as pocket probing depth (PPD), bleeding on probing (BOP), plaque index (PI), microorganism load, and species.

### 2.3. Search Strategy

Two reviewers (RZ and SX Liu) independently executed search and review of the literature to retrieve all relevant articles published up to and including May 2022. The following databases were included as electronic databases: Ovid MEDLINE, EMBASE, ScienceDirect databases, and Cochrane Central Register of Controlled Trials. A broad hand search was supplemented from the following journals: *Journal of Dental Research*; *Clinical Oral Implants Research*, *Clinical Implant Dentistry and Related Research*, *International Journal of Oral and Maxillofacial Implants*, *Journal of Periodontology*, and *Journal of Clinical Periodontology*. Finally, checking the references of all selected articles and related systematic reviews was comprised. If required, the corresponding authors were contacted and requested to provide missing data or information. In case there was any gray literature, we also searched the database System for Information on Grey literature in Europe (http://www.opengrey.eu) as recommended by high standards for systematic reviews. A commercially available software (Endnote X7; Thomson, London, UK) was used for electronic title management. Any disagreement concerning eligibility between the two reviewers during the first and second stage of the study selection was resolved by discussion or arbitration through a third reviewer (YM Liu) to reach a definitive decision.

The combination of key words from the Medical Subject Headings (MeSH) identified by an asterisk symbol (^∗^) and free text terms included: Intervention OR Therapy OR Treatment OR Mechanical debridement OR MD Professionally administered plaque removal OR PARR OR non- surgical periodontal therapy OR non-surgical therapy OR Periodontal treatment OR Periodontal therapy AND Chlorhexidine OR Chlorhexidine∗ OR Chlorhexidine phosphanilate OR Chlorhexidine gluconate OR zinc-Chlorhexidine OR chlorhexidine gluconate lidocaine hydrochloride OR CHX OR CHX formulations Probiotic treatment OR anti-microbial OR anti-infective AND Peri-implant diseases OR Peri-implant mucositis OR Mucositis^∗^ OR Peri-implant

The study inclusion and exclusion criteria are as follows:

During the first stage of the study selection, the titles and abstracts were screened and evaluated according to the following inclusion criteria: (1) English language; (2) randomized controlled clinical trial (RCT) in adult patients (>18 years); (3) assessed treatment of patients with a primary diagnosis of PiM according to standard diagnostic criteria [3]; (4) comparison of MD+CHX versus MD+placebo or MD alone; and (5) reported data in terms of clinical parameters about peri-implant mucosal inflammation (i.e., PPD, BOP, and PI) or microbial outcome.

At the second stage of the selection, all full-text articles selected in the first stage were identified according to the following exclusion criteria: (1) not RCT study design; (2) inadequate case definition; (3) inclusion of less than 10 patients; (4) received surgical treatment or other active interventions (e.g., air abrasive therapy, antibiotics therapy); (5) a follow-up assessment less than 8 weeks; (6) reported without clinical data of PiM inflammation; and (7) *in vitro* and animal model studies, case report, letters to the editor, opinion articles, review articles, interviews, and monographs.

### 2.4. Risk of Bias (Quality) Assessment

Risk of bias assessment for all the selected RCTs using the *Cochrane Handbook for Systematic Reviews of Interventions* [27] from seven criteria (random sequence generation; allocation concealment; blinding of participants and personnel; blinding of outcome assessment; incomplete outcome data; selective reporting; and other bias) was performed by two reviewers (RZ and SX Liu) independently. In general, studies were rated as “high risk of bias” (high), “low risk of bias” (low), or “unclear risk of bias” (?). Both reviewers discussed and resolved any disagreements.

#### 2.4.1. Data Items

The meta-analysis estimated diseases resolution of PiM (absence of BOP), IPPD, IBOP%, and PI% reduction after treatment as primary outcomes. Secondary outcomes included reduction of FMPPD, FMBOP%, FMPI, and changes in microorganism number and species.

#### 2.4.2. Data Synthesis

Data extraction was conducted by two blinded reviewers (RZ and SX Liu) from the included articles into predesigned data extraction template on Microsoft Excel: (1) study identification: first author's name, year of publication, journal's name and country; (2) study design (RCTs); (3) number of dental implants (4) population (subjects): sample size, gender, mean, and age range in years; (5) PiM diagnostic criteria; (6) group assessed; (7) intervention: details of CHX administration including dose, concentration, frequency, duration, any pre-treatment (mechanical or chemical disinfection) and vehicle, and oral hygiene instruction; (8) smoking habits; (9) follow-up; and (10) primary and secondary outcomes and observation period. Electronic mails were sent to respective authors in order to retrieve any relevant unpublished data that we could not extract. Any discrepancies were resolved by discussion with a third examiner (YM Liu).

#### 2.4.3. Analysis Method

Heterogeneity between RCT's meta-analysis was tested and evaluated through *Q* and *I*^2^ test. *Q* test was used to estimate the between-studies variation. When a *P* value of *Q* statistic was <0.1, it was defined as an indicator of heterogeneity. The threshold for the interpretation of *I*^2^ values was also used to estimate the heterogeneity as follows: 0–30% (low heterogeneity), 30–60% (moderate heterogeneity), >60% (substantial heterogeneity). Differences between the MD+CHX and MD alone or MD+placebo groups were expressed as weighted mean differences (WMD) and 95% confidence interval (CI) for continuous outcomes, and odds ratio (OR) and 95% CI for dichotomous outcomes, using random effect models. For continuous data, mean differences and standard errors were entered for each study. If data were not reported in terms of mean differences, the mean difference was calculated and the standard deviation was estimated using the *r*_*d*_ = sqrt (*r*_1_^2^/*n*_1_ + *r*_2_^2^/*n*_2_) formula. The meta-analysis was performed using Review Manager software (RevMan, version 5.3 for Windows).

## 3. Results

### 3.1. Study Selection

**Figure 1:**
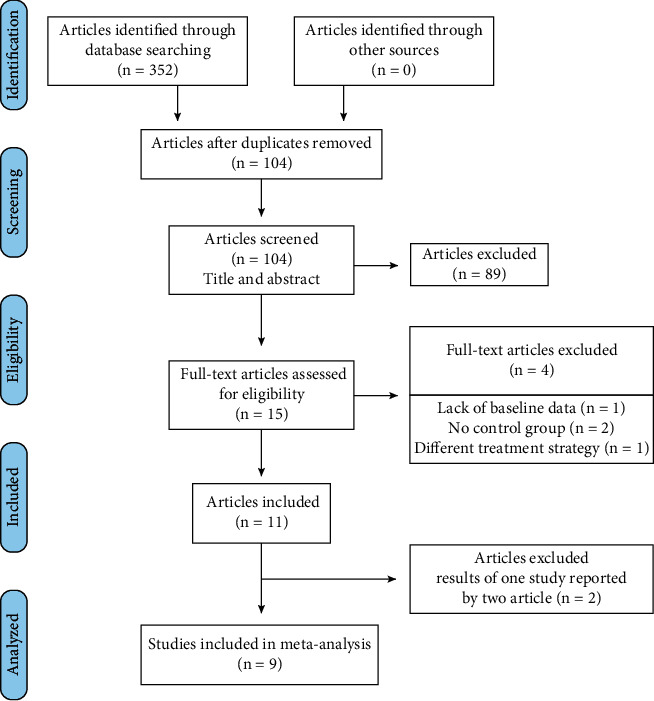
Flow chart of literature search and inclusion.

The flow diagram of the screening process is described in Figure 1. A total of 104 potentially relevant titles and abstracts were identified through the electronic and manual search. Among them, 88 articles were excluded based on the title and abstract after removing the duplicates. Therefore, 15 remaining articles were assessed for complete evaluation, but among them four were further excluded at this stage because they did not fulfill the inclusion criteria. One study conducted at the same center and on the same date was reported in two separate papers which provided clinical data of implant [23] and full mouth [29]. Similarly, Philip et al. conducted a study at Academic Centre for Dentistry Amsterdam and published two articles, respectively, about clinical changes[21] and microbiome[30]. Therefore, we combined the data of two articles and analyzed as one study for this review [21, 23].

Finally, nine studies met the inclusion criteria and were included in this systematic review [18, 20–23, 29–31].

### 3.2. Study Characteristics

Table 1 reports the general characteristics and conclusions of the 9 included studies. All of them were RCTs conducted at a single center with a parallel design and published in the English language from 2002 to 2021, spanning 19 years. The average number of participants per study was 38.5 with a minimum of 13 [24] and a maximum of 89 [21]. The average ages of patients involved in studies were range from 41.5 to 70 years old. The follow-up period of included studies ranged from 3 months [13, 18, 19, 21, 22, 31] to 12 months [23].

### 3.3. Treatment Modalities

Oral hygiene instructions were provided in all the studies. Nonsurgical mechanical therapy was performed with ultrasonic devices and polishing at baseline. Prescribed CHX varied in types (mouth rinses, gels, spray, and irrigation devices), concentration (0.03%, 0.06%, 0.12%, 0.2%, 0.5%, and 1%), frequency (once or twice a day), and period of intake (10 or 14 days, 4 or 12 weeks, and 1 year). As the application of CHX gel was always combined with CHX mouth rinse [24], we did the subgroup analysis according to oral irrigation and mouth rinse/gel in meta-analysis. The types of administration were as follows: three trials used mouth rinse [13, 21, 23], two trails used gel [19, 31], one trail used irrigation [18], and three trails used both irrigations and mouth rinse [20, 22, 24]. All studies were placebo-controlled except one study [22].

Four studies included only nonsmokers or former smokers [13, 18, 20, 22], and five studies included both nonsmokers and smokers and reported the constituents of different smoking types [19, 21, 23, 24, 31]. Number of subjects, six distribution, and mean age in each group were always reported (Table 1).

### 3.4. Risk of Bias

**Figure 2:**
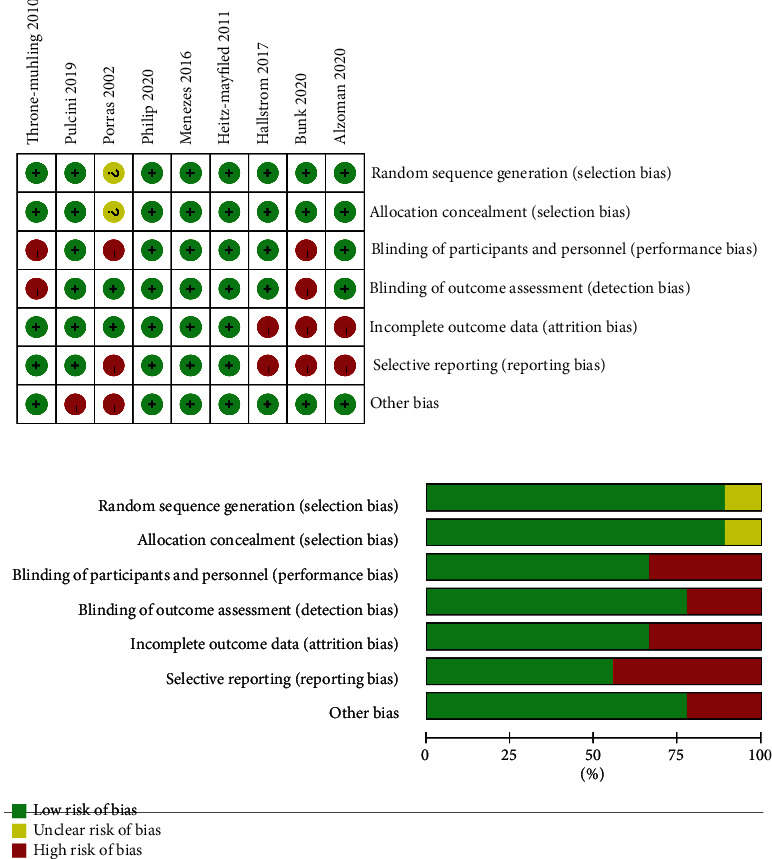
Quality assessment of the selected studies (The Cochrane Collaboration tool for assessing risk of bias).

The results of the risk of bias assessment within studies are depicted in Figure 2. Though we tried our best to contact corresponding authors of included studies to seek information according to the advice in Section 16.1.2 of the Cochrane Handbook, no response was obtained. Only one study did not describe the process of randomization and allocation concealment [22]. Porras et al. [22] did not use placebo in the control group; thus, the blinding to the participants cannot be achieved. Two studies did not explain the binding of outcome assessment [18, 24]. The study of Alzoman et al. [13], Hallstrom et al. [31], and Bunk et al. [18] did not report the mean and standard deviation of IPPD or IBOP%, which lead to an incomplete outcome data. Three studies included in this systematic review were considered with a low risk of bias.

### 3.5. Study Outcomes

In detail, the main outcomes and statistical differences between the test group (MD+CHX) and control group (MD+placebo/MD alone) were described and summarized in Table 1.

### 3.6. Disease Resolution

**Figure 3:**
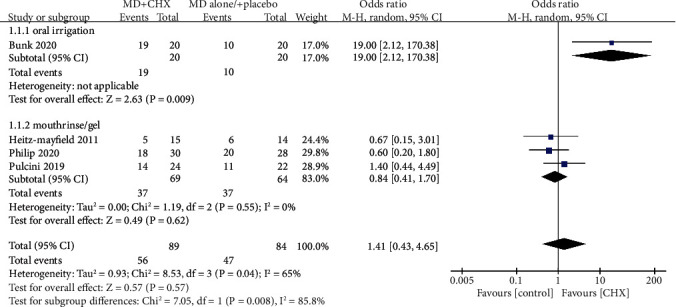
Forest plot of disease resolution of PiM.

Absence of BOP-positive site around implant was the sign of achieving complete disease resolution [21], and there were four studies reported the results of diseases resolution at final visit. Bunk et al. reported more percentage of disease resolution in the CHX group (95%) than the control group (50%) at 12 weeks (*P* < 0.05). On the contrary, three studies [21, 23, 24] found similar disease resolution percentage between two groups. Overall diseases resolution rates of PiM were 62.92% with CHX and 55.95% with control, which did not differ significantly (OR = 1.41 (95% CI (0.43, 4.65), *P* = 0.57)). Interstudy heterogeneity appeared significant regarding disease resolution (*P* < 0.05, *I*^2^ = 65%). Hence, subgroup analysis was conducted and could explain heterogeneity based on variation in CHX types (Figure 3). The results indicated that oral irrigation of CHX may have more benefits on the resolution of PiM.

### 3.7. PPD Reduction

Pocket probing depth around the implant was evaluated by seven studies at different time intervals. In the study of Porras et al., as the control group showed greater IPPD than the test group at the baseline, so the reduction of IPPD of the control group was significantly greater compared to the test group at 3 months. Five studies showed a significant reduction of IPPD in both the test and control groups and did not highlight the differences between them [19–21, 23, 24]. Only one study reported the IPPD of the test group was significantly lower than control group at both 3, 6, and 12 weeks of follow-up [13]. Mean IPPD reduction regarding MD+CHX treatment at the end of observation period ranged from 0.36 (±0.6) [20] to 2.11 (±0.31) mm [13], while this reduction ranged from 0.23 (±0.68) [20] to 2.17 (±0.24) mm [13] for control.

**Figure 4:**
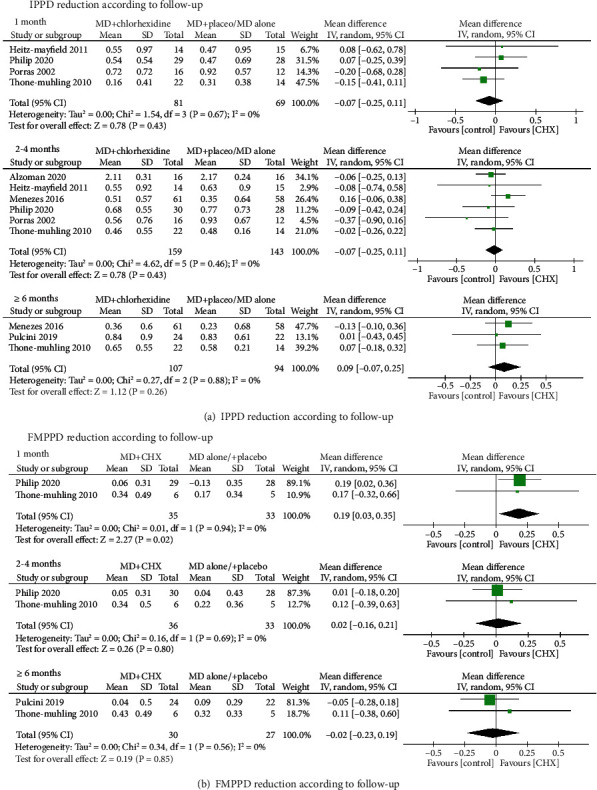
Forest plot of PD reduction (a) at implant level and (b) at full-mouth level.

Four studies [19, 21, 22, 24] reported IPPD reduction at 1 month after treatment and the WMD in IPPD reduction between the experimental and control groups amounted to -0.07 mm (95% CI (-0.25, 0.11), *P* = 0.43) not favoring the additional CHX therapy (*P* value for heterogeneity: 0.67, *I*^2^ = 0% = low heterogeneity) (Figure 4(a)). Similar results were found at longer term and the WMD of -0.02 mm (95% CI (-0.26, 0.22), *P* = 0.84) (*P* value for heterogeneity: 0.46, *I*^2^ = 0% = low heterogeneity) for 2-4 months and the WMD of 0.09 mm (95% CI (-0.07, 0.25), *P* = 0.26) (*P* value for heterogeneity: 0.88, *I*^2^ = 0% = low heterogeneity) for more than 6 months follow-up.

At full mouth level, the full mouth pocket probing depth (FMPPD) were recorded by three studies [21, 24, 29] and meta-analysis were conducted at different time points. More FMPPD reduction was observed following CHX adjunctive therapy at 1 month with the WMD of 0.19 mm (95% CI (0.03, 0.35, *P* = 0.02) (*P* value for heterogeneity: 0.94, *I*^2^ = 0% = low heterogeneity) (Figure 4(b)), whereas no significant difference was found at 2-4 months (0.02, 95% CI (-0.16,0.21), *P* = 0.8) and ≥6 months (-0.02, 95% CI (-0.23,0.19), *P* = 0.85) between the experimental and control groups. The interstudy heterogeneity was both low, given an *I*^2^ of 0%.

### 3.8. BOP Changes

Implant bleeding on probing sites (IBOP%) were expressed as percentage of sites with bleeding of the total number of available sites and evaluated by eight studies [13, 18–21, 23, 24, 31]. Three studies [13, 22, 23] showed a significant difference of IBOP% reduction in favor of the MD+CHX treatment. However, the other studies did not report differences between groups. Mean IBOP% reduction regarding the MD+CHX treatment at the end of observation period ranged from 6 (±10) [24] to 57.5 (±25.75) [18], while this reduction ranged from 0 (±21) [24] to 45 (±28.75) [18] for control.

**Figure 5:**
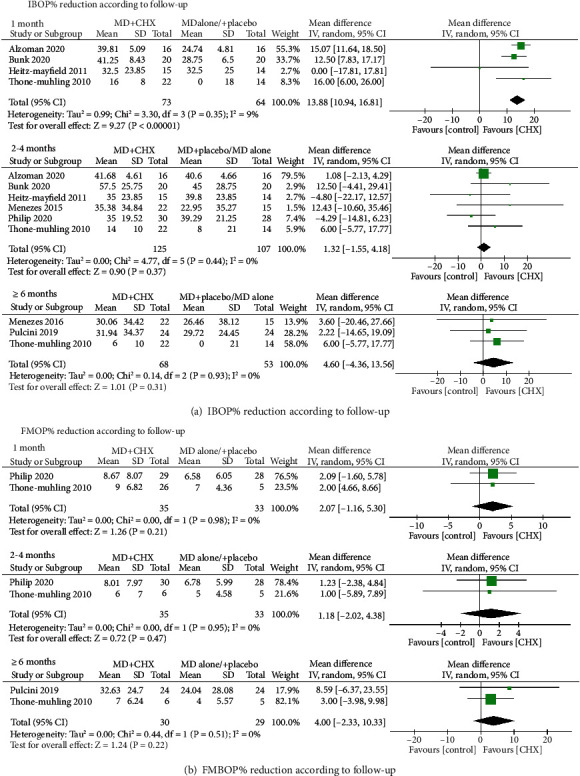
Forest plot of BOP% reduction (a) at implant level and (b) at full-mouth level.

At 1-month follow-up, four studies were included to conduct meta-analysis and the WMD in IBOP% reduction between the experimental and control groups amounted to 13.88 (95% CI (10.94, 16.81), *P* < 0.00001) (*P* value for heterogeneity: 0.35, *I*^2^ = 9% = low heterogeneity) (Figure 5(a)). So, there was greater IBOP% reduction with MD+CHX indicating adjunctive CHX treatment was effective at 1 month. However, at 2-4 months (1.32, 95% CI (-1.55, 4.18), *P* = 0.37) and ≥6 months (4. 6, 95% CI (-4.36, 13.55), *P* = 0.31) of follow-up, the experimented group presented similar IBOP% changes with control group. The interstudy heterogeneity was both low, given an *I*^2^ of 0%.

At full mouth level, the full mouth bleeding on probing sites (FMBOP%) were recorded by three studies[21, 24, 29] and meta-analysis were conducted at different time points. The meta-analysis failed to show a significant FMBOP% reduction at both 1 month (2.07, 95% CI (-1.16, 5.3), *P* = 0.21), 2-4 months (1.18, 95% CI (-2.02, 4.38), *P* = 0.47) and ≥6 months (4.95% CI (-2.33, 10.33), *P* = 0.22) follow-up between MD+CHX and control (Figure 5(b)). The interstudy heterogeneity was both low, given an *I*^2^ of 0%.

### 3.9. PI Changes

Six studies performed the measurement of the implant plaque index (IPI) around the selected implants [13, 18, 20, 21, 23, 24]. Bunk et al. found the change of IPI seems to be highly dependent upon IPI measured at baseline. But, in the studies of Menezes et al. and Pulcini et al., the IPI of the control group were higher than the test group at baseline. Mean IPI reduction regarding MD+CHX treatment at the end of observation period ranged from 0.01 (±0.03)[24] to 0.46 (±0.48)[18], while this reduction ranged from 0.1 (±0.16)[13] to 0.4 (±0.28) [20] for control.

**Figure 6:**
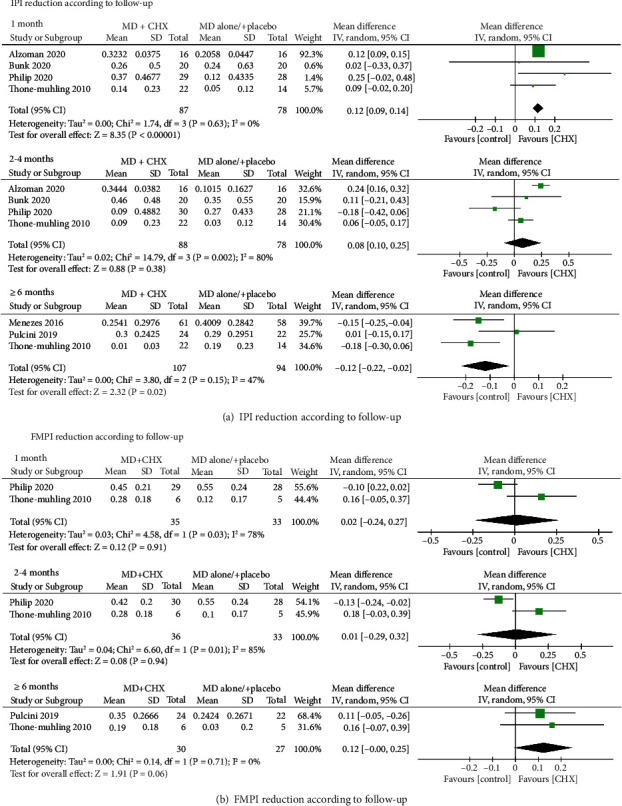
Forest plot of PI reduction (a) at implant level and (b) at full-mouth level.

At 1-month follow-up, based on four studies, the WMD in IPI reduction between the experimental and control groups amounted to 0.12 (95% CI (0.09, 0.14), *P* < 0.00001) favoring the additional CHX therapy (*P* value for heterogeneity: 0.63, *I*^2^ = 0% = low heterogeneity) (Figure 6(a)). But no significant difference was observed at 2-4 months (0.08, 95% CI (-0.1,0.25), *P* = 0.38) (*P* value for heterogeneity: 0.002, *I*^2^ = 80% =  substantial heterogeneity). Conversely, when evaluating IPI reduction at ≥6 months follow-up, control group demonstrated a significant greater IPI reduction than MD+CHX group(-0.12, 95% CI (-0.22,-0.02), *P* = 0.02) (*P* value for heterogeneity: 0.15, *I*^2^ = 47% =  moderate heterogeneity).

At full mouth level, the full mouth plaque index (FMPI) was recorded by three studies [21, 24, 29] and meta-analysis were conducted at different time points. The heterogeneity between trials was high except for at ≥6 months follow-up (*P* = 0.71, *I*^2^ = 0% = low heterogeneity) (Figure 6(b)). The meta-analysis failed to show a significant difference regarding FMPI reduction between the MD+CHX and control groups at all time points.

### 3.10. Microbiological Outcomes

Five studies [19, 22–24, 30] performed the collection of the biological samples in the deepest peri-implant pockets (Table 2). The plaque samples were all collected from subgingival plaque except for Porras et al. [22] which collected from supragingival plaque. Studies used sterile paper points for 10 s [22, 23] and 20 s [24] in the peri-implant pocket. Only Pulcini et al. [23] reported the time between the collection and processing of the samples.

Among five studies, different techniques were applied to investigate the microbiological outcomes, including DNA probes, RT-qPCR, DNA-DNA hybridization, quantitative (CFU), and16S rRNA sequencing. So, a meta-analysis could not be performed due to the different types of microbiological results. Thone-Muhling et al. [24] and Heitz-Mayfield et al. [19] found there were no significant differences in mean total DNA counts between test and control groups (*P* > 0.1).

### 3.11. Adverse Events

Any side effects or adverse events during adjunctive CHX application were recorded in four studies [19, 21, 24, 31]. No adverse events were reported except one study. Philip et al. reported staining of the teeth or tongue and taste alteration in the CHX group [21].

## 4. Discussion

CHX was always regularly considered and recommended for individuals who are at several different stages during dental implant treatment, including presurgical mouth rinse, postoperative protocols, and during implant maintenance [14]. CHX could damage the cellular membranes which was a broad-spectrum antimicrobial agent [17]. Therefore, many dentists recommended the regular CHX application during implant inflammation. Currently, the guidelines even suggest that management of peri-implant diseases could include nonsurgical debridement with carbon fiber or plastic curettes and irrigate the pocket with 0.2% CHX [32]. However, some relatives RCTs indicated no adjunctive benefits of CHX in the treatment of PiM. Therefore, the primary aim of this study was to evaluate whether supplementation of CHX with nonsurgical therapy resulted in improved outcomes in the management of peri-implant mucositis.

Our results of this meta-analysis support the no adjunctive clinical benefits in terms of disease resolution and IPPD reduction at both short-, medium-, and long-term of follow-up evaluation. It has been clearly demonstrated that the CHX could confer some clinical benefit in the managing of gingivitis [33, 34]. However, the efficacy of adjunctive CHX treatment seems dispensable as the cure rate of PiM did not improve. Based on our data, the resolution of inflammation was not achieved in all patients with PiM. Compared with periodontal tissue, the peri-implant tissue seems more susceptible by many factors, such as absence of keratinized gingival, the lack of periodontal ligament and Sharpey's fibers, and the presence of residual cement [35], which may limit the access to oral hygiene control and plaque control [23]. Moreover, both animal and human experiments have demonstrated the significant quantitative and qualitative differences of supracrestal connective tissue compartment around the teeth and dental implants in regard to the number of collagen fiber orientation, fibroblasts, and vascular supply [29]. A systematic review focusing on whether CHX improves outcomes in the management of peri-implant diseases was conducted by Liu et al., and only four studies (reported from 2002 to 2016) were included [25]. In addition, the previous meta-analysis only analyzed the outcomes of IPPD changes and did not find a significant difference between CHX+MD and placebo+MD/MD alone which was consistent with our results of meta-analysis.

Interestingly, our subgroup analysis indicated that oral irrigation of CHX may have more benefits than CHX mouth rinse or CHX gel on the resolution of PiM. The ideal treatment of PiM was achieving the complete resolution of diseases. However, based on the results of the present systematic review, the oral irrigation with CHX seems achieved a higher PiM resolution rate (95%) compared with rinsing with CHX solution or CHX gel (53.62%). Oral irrigator, also known as dental water jet or water flosser, an electric device which has been available for just over fifty years and delivers pulsating fluid through controlled pressure to provide the compression and decompression of gingival tissue, removing supragingival plaque and flushing out subgingival bacteria and other debris [36, 37]. Oral irrigators have often been used in addition to tooth brushing and shown to be effective in reducing oral biofilm, clinical periodontal indexes, and host inflammatory mediators by reducing proinflammatory cytokines (IL-1*β* and PGE2) in the gingival crevicular fluid [38, 39]. Tutuncuoglu et al. assessed the efficacy of oral irrigation in PiM patients and concluded that the use of an oral irrigator can be as effective as an interdental brush in interdental cleaning [40]. Consistent with our meta-analysis, oral irrigation of CHX would result in better plaque control and better resolution of PiM, compared with other types such as CHX mouth-rinse or CHX gel.

In term of other primary outcomes, the magnitude of the reduction in IPPD varied among the included studies. Five studies revealed a decrease in IPPD that was generally <1 mm and only one study reported a IPPD reduction to 2 mm in both the test and control groups. Data synthesis of the included studies evaluated that WMD in IPPD reduction at different time points and were both not in favor of the additional CHX therapy over MD alone. And regarding the BOP changes, greater IBOP% reduction of the test group was only found at 1-month follow-up, indicating adjunctive CHX treatment was effective on the inflammation control of peri-implant tissue at short term. Similar changes were also found of PI reduction around peri-implants. Conversely, the control group demonstrated a significant greater IPI reduction at ≥6 months follow-up. Evidences indicated that significant short-term improvements of plaque control around implants by adjunctive CHX treatment. CHX is also often advised for short-term use only (2-4 weeks).

As an antiseptic mouthwash or irrigation, CHX solution has a full-mouth antimicrobial effect on bacteria, fungus, and virus causative for various of different oral infectious diseases, such as gingivitis, periodontitis, and caries [17]. In our study, more FMPPD reduction were observed following CHX adjunctive therapy at 1 month compared with the control group. Other benefits of full-month parameters failed to observed. Regarding the small number of included studies, limited data available. and the variability of CHX application, these factors may be explained by these limitations. The microbiological outcomes of oral bacteria were reported in five studies but meta-analysis could not be performed due to the variability and different types of microbiological results [19, 21–24]. Three studies showed no significant differences of microbial outcomes between groups [19, 23, 24]. Philip et al. used 16S V4 rRNA gene amplicon sequencing to analyzed bioinformatically and found peri-implant sites with mucositis harbor ecologically less complex and less anaerobic biofilms with lower biomass than patient-matched dental sites with gingivitis while they elicit an equal inflammatory response [30]. So distinct from gingivitis, more aerobic bacteria such as Neisseria and Haemophilu were survived in PiM-related plaque community. They also found that the inflamed implant sites had a lower plaque index than the dental sites with gingivitis, indicating the inflammatory response around the implants is triggered by the presence and characteristics of the implant (both its structure and material) and not the oral microflora. These aspects of PiM may help to explain the minor microbiocidal changes of locally delivered CHX as an adjunct to MD compared to the control.

In recent years, several adjunctive or alternative therapies (such as antiseptic and antibiotic therapy, probiotics, photodynamic treatment) to MD have already been applied and evaluated in order to gain better control of the progression of the PiM. However, no beneficial effect in resolving peri-implant mucositis was found of these therapies [41, 42]. Therefore, the primary aim of this study was to evaluate whether supplementation of CHX with nonsurgical therapy resulted in improved outcomes in the management of PiM. Additionally, according to what we know, this is the first systematic review that discusses the preexisting criteria of CHX application (oral irrigator and mouth rinse/gel) for PiM. Our results also support that CHX adjunctive therapy cannot bring evident clinical benefits compared to MD alone in the treatment of PiM. In contrast to the gingivitis, up to now, there was no evidence for the primary prevention, plaque control or complete of PiM [43]. It seems the vital item of peri-implant health was the prevention and control of inflammation. Therefore, any risk factors for the development of PiM should be given attention. An environmental, behavioral, or biological factor that if present directly increases the probability of PiM should be avoided if possible, such as excess cement, smoking, ideal design, and surface characteristics of transmucosal portion of implants.

However, owing to the inevitably differences between included studies, the present study has a few limitations. At first, included studies demonstrated some variability in the type of CHX used, dose, and method of administration. We only conducted subgroup analysis between oral irrigator and mouth rinse/gel of CHX. For other clinical variables, subgroup analysis was not performed because they not have enough power to detect a true effect with fewer studies. Considering these may increase the clinical heterogeneity of the study, we used random-effects model to minimize the statistical error. *I*^2^ statistics showed a low level of heterogeneity in terms of PPD reduction, and BOP% changes, suggesting the heterogeneity of the data was acceptable. However, the heterogeneity of disease resolution was high. Hence, subgroup analysis was conducted and could explain heterogeneity based on variation in CHX types. Well-designed large clinical trials are needed in future to directly investigate the effects of additional CHX application on PiM. Second, as smoking was demonstrated a risk factor of peri-implant diseases, only four studies included only nonsmokers or former smokers [13, 18, 20, 22]. The other five studies included both nonsmokers and smokers, and reported the constituents of different smoking. Third, some included studies reported the primary outcomes with different parameters and measuring method, such as the information about BOP, plaque control, or microbiological load. In order to obtain more useful and adequate data, Alzoman, Bunk, and Heitz-Mayfield were contacted, but none of them replied. Finally, because of the high heterogeneity, the limited available data of the included studies, and the small size of the studies analyzed in our review, the quality of the evidence might be decreased, and the impact of the conclusions of this meta-analysis could be reduced.

## 5. Conclusion

Adjunctive CHX application may have some benefits to improve the efficacy of MD in PiM treatment by reducing IBOP%, IPI, and FMPPD in short-term. But these benefits were disappeared at medium- and long-term follow-up. In order to achieve better disease resolution of PiM, adjunctive CHX irrigation with MD may be suggested and has positive potential. Well-designed large clinical trials are needed in future.

## Figures and Tables

**Table 1:** Characteristics of the included studies.

Study journal region	Type	Number of implants	Clinical parameters	Subjects M/F age	Peri-implant mucositis definition	Treatment	CHX administration	Smoking	Follow-up	Mean (SD) outcome
Porras et al. [22] *J Periodontol* American	RCT single-blind parallel	28	IPPD, ICAL, microbiological parameters	16 NG 58.9	BOP + plaque + PD ≤ 5 mm + incipient radiographic lesion	With OHI, MD alone (control), MD+ CHX (test)	Local irrigation with 0.12% CHX and topical application of CHX gel were conducted after MD. 0.12% CHX mouthrinse twice a day for 10 days.	No	3 months (days 30, 90)	Test: IPPD: 3.27 (SD:0.81) (BL) to 2.71 (SD: 0.7) (3 months) (sign.) ICAL: 2.63 (SD:1.6) (BL) to 2.3 (SD:1.5) (3 months) Control: IPPD: 3.48 (SD: 0.61) (BL) to 2.55 (SD: 0.72) (3 months) (sign.) ICAL: 3.08 (SD: 1.27) (BL) to 2.01 (SD: 1.23) (3 months) (sign.)

Thone-Muhling et al. [24] *Clin Oral Impl Res* Germany	RCT single-blind parallel	36	IPPD, FMPPD, IGR, FMGR, ICAL, FMCAL, IBOP%, FMBOP%, IGI, FMGI, IPI, FMPI	13 8 : 5 51.5	BOP and/or GI ≥ *I*+ no evidence of radiographic bone loss	With OHI, MD alone (control), MD+CHX (test)	Topical application of I% CHX gel once and brush at dorsal of tongue; tonsil was sprayed with 0.2% CHX spray once day and 0.2% CHX mouthrinse twice daily for 14 days.	38%	8 months (days 30, 60, 120, 240)	Test: IPPD: 3.49 (SD: 0.78) (BL) to 3.03 (SD: 0.46) (4 months) FMPPD: 2.4 (SD: 0.48) (BL) to 2.28 (SD: 0.4) (4 months) IGR: 0.21 (SD: 0.25) (BL) to 0.25 (SD: 0.37) (4 months) FMGR: 0.78 (SD: 0.55) (BL) to 0.81 (SD: 0.57) (4 months) ICAL: 3.7 (SD: 0.72) (BL) to 3.29 (SD: 0.38) (4 months) FMCAL: 3.19 (SD: 0.72) (BL) to 3.08 (SD: 0.49) (4 months) IBOP%: 22 (SD: 11) (BL) to 8 (SD: 9) (4 months) (sign.) FMBOP%: 9 (SD: 9) (BL) to 7 (SD: 7) (4 months) IGI: 0.6 (SD: 0.24) (BL) to 0.23 (SD: 0.23) (4 months) FMGI: 0.33 (SD: 0.19) (BL) to 0.18 (SD: 0.13) (4 months) (sign.) IPI: 0.02 (SD: 0.04) (BL) to 0.02 (SD: 0.04) (4 months) FMPI: 0.18 (SD: 0.07) (BL) to 0.17 (SD: 0.09) (4 months) Control: IPPD: 3.4 (SD: 0.62) (BL) to 2.92 (SD: 0.63) (4 months) (sign.) FMPPD: 2.36 (SD: 0.39) (BL) to 2.19 (SD: 0.34) (4 months) IGR: 0.33 (SD: 0.42) (BL) to 0.36 (SD: 0.47) (4 months) FMGR: 1.13 (SD: 0.5) (BL) to 1.26 (SD: 0.55) (4 months) ICAL: 3.73 (SD: 0.38) (BL) to 3.28 (SD: 0.38) (4 months) (sign.) FMCAL: 3.48 (SD: 0.31) (BL) to 3.45 (SD: 0.49) (4 months) IBOP%: 17 (SD: 19) (BL) to 8 (SD: 9) (4 months) FMBOP%: 5 (SD: 4) (BL) to 5 (SD: 4) (4 months) IGI: 0.62 (SD: 0.36) (BL) to 0.34 (SD: 0.24) (4 months) FMGI: 0.29 (SD: 0.11) (BL) to 0.18 (SD: 0.08) (4 months) (sign.) IPI: 0.01 (SD: 0.02) (BL) to 0.13 (SD: 0.3) (4 months) FMPI: 0.15 (SD: 0.07) (BL) to 0.2 (SD: 0.13) (4 months)

Heitz-Mayfield et al. [19] *Clin Oral Impl Res* Australia	RCT placebo double-blind parallel	29	IBOP-positive sites, IPPD, total DNA count	29 14 : 15 69	BOP+no evidence of radiographic bone loss	With OHI, MD+placebo (control), MD+CHX (test)	Dental gel containing 0.5% CHX for 4 weeks	50%/33%	3 months (days 30.90)	Test: IPPD: 3.67 (SD: 0.92) (BL) to 3.12 (SD: 0.92) (3 months) Total DNA count: 5.24 (SD: 0.5) (BL) to 5.31 (SD: 0.48) (3 months) Control: IPPD: 3.6 (SD: 0.95) (BL) to 2.97 (SD: 0.85) (3 months) Total DNA count: 5.44 (SD: 0.37) (BL) to 5.09 (SD: 0.53) (3 months)

Menezes et al. [20] *J Periodontal* Brazil	RCT placebo double-blind parallel	119	IPI%, IGBI%, IPPD, IBOP%, IKM width	50 42/58 55.96/61.16	BOP+no radiographic signs of bone loss	With OHI, MD+placebo (control), MD+CHX (test)	CHX solutions applied to brush dorsum of tongue, subgingival irrigation and rinsing twice daily for 14 days.	No	6 months (days 30, 90, 180)	Test: IPI%:38.52 (SD: 34.02) (BL) to 10.24 (SD: 20.09) (3 months) (sign.) IGBI%: 36.88 (SD: 32.47) (BL) to 10.24 (SD: 22.07) (3 months) (sign.) IPPD: 2.85 (SD: 0.6) (BL) to 2.34 (SD: 0.54) (3 months) (sign.) IBOP%: 75.82 (SD: 33.98) (BL) to 40.44 (SD: 35.64) (3 months) (sign.) IKM width: 1.61 (SD: 1.76) (BL) to 1.29 (SD: 1.71) (3 months) (sign.) Control: IPI%: 52.15 (SD: 32.2) (BL) to 13.79 (SD: 25.72) (3 months) (sign.) IGBI%: 28.01 (SD: 32.47) (BL) to 9.48 (SD: 14.68) (3 months) (sign.) IPPD: 2.72 (SD: 0.68) (BL) to 2.37 (SD: 0.6) (3 months) (sign.) IBOP%: 67.54 (SD: 34.38) (BL) to 44.59 (SD: 36.1) (3 months) (sign.) IKM width: 1.68 (SD: 1.61) (BL) to 1.74 (SD: 1.56) (3 months) (sign.)

Hallstrom et al. [31] *Int J Dent Hygiene* Sweden	RCT placebo double-blind parallel	37	FMPI%, FMBOP%, FMPPD%, IPPD%	37 20 : 17 69	PD ≥ 4 mm and/or BOP + CBL < 2 mm	With OHI, MD+placebo (control), MD+CHX (test)	Dental gel containing 0.2% CHX digluconate, once daily for 12 weeks	65%	3 months (days 28, 84)	Test: FMPI%: 27 (BL) to 20 (3 months) (sign.) FMBOP%: 18 (BL) to 14 (3 months) FMPPD%: 5 (BL) to 4 (3 months) IPPD%: 69 (BL) to 33 (3 months) (sign.) Control: FMPI%: 23 (BL) to 20 (3 months) (sign.) FMBOP%: 18 (BL) to 14 (3 months) FMPPD%: 6 (BL) to 4 (3 months) IPPD%: 70 (BL) to 55 (3 months)

Pulcini et al. [23] *J Clin Periodontol/* *Bollain et al. [*29*] Clin Oral Invest* Spain	RCT placebo double-blind parallel	One or more per subject	PI, IBOP%, IPPD, CLI, microbiological parameters, FMPI, FMBOP%, FMPPD, staining	54 27 : 27 61.15	BOP and/or suppuration+no evidence of radiographic bone loss	With OHI+airpolishing, MD+placebo (control), MD+CHX (test)	0.03% CHX and 0.05% CPC mouthrinse, twice daily for 1 year	7.4%/14.8%	12 months (6, 12 months)	Test: IBOP%: 58.64 (SD: 27.49) (BL) to 10.42 (SD: 13.74) (12 months) (sign.) IPI: 0.48 (SD: 0.26) (BL) to 0.18 (SD: 0.22) (12 months) (sign.) IPPD: 3.36 (SD: 0.78) (BL) to 2.5 (SD: 0.43) (12 months) (sign.) CLI: 8.63 (SD: 1.97) (BL) to 9.1 (SD: 1.86) (12 months) FMPI: 0.52 (SD: 0.3) (BL) to 0.178 (SD: 0.14) (12 months) (sign.) FMBOP%: 29.95 (SD: 12.3) (BL) to 5.5 (SD: 5.75) (12 months) (sign.) FMPPD: 2.45 (SD: 0.56) (BL) to 2.41 (SD: 0.4) (3 months) Control: IBOP%:46.3 (SD: 24.17) (BL) to 14.39 (SD: 18.04) (12 months) (sign.) PI: 0.54 (SD: 0.3) (BL) to 0.25 (SD: 0.29) (3 months) (sign.) IPPD: 3.38 (SD: 0.6) (BL) to 2.57 (SD: 0.57) (12 months) (sign.) CLI: 9.45 (SD: 2.19) (BL) to 10.09 (SD: 2.15) (12 months) FMPI: 0.48 (SD: 0.3) (BL) to 0.23 (SD: 0.21) (12 months) (sign.) FMBOP%: 27.65 (SD: 9.6) (BL) to 7.98 (SD: 7.55) (12 months) (sign.) FMPPD: 2.63 (SD: 0.26) (BL) to 2.54 (SD: 0.32) (3 months)

Bunk et al. [18] *Clin Oral Impl Res* Germany	RCT placebo single-blind parallel	40	IPI, IBOP-positive sites, prevalence of PiM, mucositis Severity score	40 17 : 23 70	BOP and/or suppuration+no evidence of radiographic bone loss	With OHI, MD alone (control), MD+CHX (test)	0.06% CHX oral irrigation, once daily for 12 weeks	No	3 months (days 28, 56, 84)	Test: IBOP-positive sites: 2.4 (SD: 0.88) (BL) to 0.1 (SD: 0.45) (3 months) (sign.) mucositis severity score: 9.05 (SD: 1.79) (BL) to 2.1 (SD: 2.22) (3 months) (sign.) IPI: 1.26 (SD: 0.4) (BL) to 0.79 (SD: 0.6) (3 months) (sign.) prevalence of PiM: 100% (BL) to 5% (3 months) (sign.) Control: IBOP-positive sites: 2.35 (SD:0.99) (BL) to 0.85 (SD: 1.09) (3 months) (sign.) mucositis severity score: 9.05 (SD: 2.54) (BL) to 4.5 (SD: 3.27) (3 months) (sign.) IPI: 1.33 (SD: 0.52) (BL) to 0.83 (SD: 0.63) (3 months) (sign.) Prevalence of PiM: 100% (BL) to 50% (3 months, implant level) (sign.)

Alzoman et al. [13] *Oral Hlth Prev dent* Saudi Arabia	RCT placebo double-blind parallel	32		32 20 : 12 41.25	BOP and/or erythema, swelling, suppuration pus+CBL < 2 mm	With OHI MD+placebo (control), MD+CHX (test)	0.12% CHX mouthrinse, twice daily for 2 weeks	No	3 months (days 21, 42, 84)	Test: IPI: 0.42 (SD: 0.02) (BL) to 0.07 (SD: 0.04) (3 months) (sign.) IBOP%: 51.1 (SD: 0.5) (BL) to 9.42 (SD: 4.84) (3 months) (sign.) IPPD:4.2 (SD: 0.3) (BL) to 2.09 (SD: 0.32) (3 months) (sign.) Control: IPI: 0.43 (SD: 0.04) (BL) to 0.33 (SD: 0.04) (3 months) IBOP%: 48.7 (SD: 1.3) (BL) to 36.4 (SD: 4.84) (3 months) IPPD:4.1 (SD: 0.3) (BL) to 3.37 (SD: 0.5) (3 months)

Philip et al. [21] J Clin Periodontol/Philip et al. [30] J Clin Periodontol Netherlands	RCT placebo double-blind parallel	One or more per subject	IPI, IBI, IBOP%, IPPD, FMPI, FMGI, FMBOP%, FMPPD, percentages of completed patients, microbiological parameters	89 48/41 61.87	BOP and/or erythema, swelling, suppuration pus +CBL < 2 mm	With OHI, MD+placebo (control), MD+CHX (test)	0.2% CHX mouthrinse, twice daily for 1 month	13%/11%	3 months (days 30, 90)	Test: IPI: 0.61 (SD: 0.54) (BL) to 0.52 (SD: 0.41) (3 months) IBI: 1.03 (SD: 0.44) (BL) to 0.28 (SD: 0.3) (3 months) (sign.) IBOP%: 43.88 (SD: 22.52) (BL) to 8.88 (SD: 12.17) (3 months) (sign.) IPPD: 3.44 (SD: 0.6) (BL) to 2.76 (SD: 0.47) (3 months) (sign.) FMPI: 0.5 (SD: 0.23) (BL) to 0.08 (SD: 0.07) (3 months) (sign.) FMGI: 0.32 (SD: 0.33) (BL) to 0.36 (SD: 0.23) (3 months) FMBOP%: 10.27 (SD: 8.82) (BL) to 2.26 (SD: 2.14) (3 months) (sign.) FMPPD: 2.67 (SD: 0.31) (BL) to 2.62 (SD: 0.3) (3 months) Control: IPI: 0.6 (SD: 0.5) (BL) to 0.33 (SD: 0.25) (3 months) (sign.) IBI: 1.08 (SD: 0.52) (BL) to 0.19 (SD: 0.32) (3 months) (sign.) IBOP%: 47.02 (SD: 24.45) (BL) to 7.73 (SD: 13.96) (3 month) (sign.) IPPD: 3.17 (SD: 0.78) (BL) to 2.4 (SD: 0.67) (3 month) (sign.) FMPI: 0.61 (SD: 0.27) (BL) to 0.06 (SD: 0.08) (3 months) (sign.) FMGI: 0.3 (SD: 0.41) (BL) to 0.29 (SD: 0.19) (3 months) FMBOP%: 8.64 (SD: 6.89) (BL) to 1.86 (SD: 2.98) (3 months) (sign.) FMPPD: 2.46 (SD: 0.38) (BL) to 2.5 (SD: 0.47) (3 months)

**Table 2:** Microbiological methods of the selected studies.

Study	Sampling type	Instrument collection	Load implant collection	Time	Transport media/processing	Technique	Targeted oral bacteria	Major findings
Porras et al. [22]	Supragingival plaque	Sterile paper points	Deepest PPD	10s	In a sterile plastic container/WD	DNA probes	*A. actinomycetemcomitans, P. intermedia, P. gingivalis, E. corrodens, C. retus, B. forsythus, T. denticola, F. nucleatum*	Most of the sites were free of pathogens with the exception of *E. corrodens* after both treatment at 3 months.
Thone-Muhling et al. [24]	Subgingival plaque	Sterile paper points	Deepest PPD	20s	In a sterile Eppendorf tube/WD	RT-qPCR	*A. actinomycetemcomitans, P. intermedia, P. gingivalis, D. pneumosintes, C. retus, P. micra*	The microbiological outcomes showed no significant reductions for implants and teeth in the total bacterial load after 8 months. In both groups, a decrease in the bacterial counts was detected after 24 h, although not significant for all bacteria and for all groups.
Heitz-Mayfield et al. [19]	Subgingival plaque	Sterile paper points	Deepest PPD	WD	In a sterile Eppendorf tube/WD	DNA-DNA hybridization	40 subgingival species with the additional of *Staphylococcus aureus*	There were no significant differences in mean total DNA counts between test and control group. (*P* > 0.1)
Pulcini et al. [23]	Subgingival plaque	Sterile paper points	Deepest PPD	10s	In a screw-capped vial/within 2 h	Quantiative (CFU)	*P. gingivalis, P. intermedia, T. forsythia, P. micra, C. retus, F. nucleatum, Capnocytophaga* spp.*, E. corrodens*	No significant differences between groups were observed at any time point in regards to the frequency of detection of target species. For proportions of target species, the test group showed statistically significant reductions in the proportions of *Porphyromonas gingivalis* after 3 months and of *Prevotella intermedia*, *F. nucleatum* and *P. micra* up to 6 months (*P* < 0.05). In the control group, significant reductions were only observed for *F. nucleatum* after 6 months (*P* = 0.02).
Philip et al. [21]	Subgingival plaque	Sterile implant deplaquer	Deepest PPD	WD	In a sterile Eppendorf tube/WD	16S rRNA sequencing	Subgingival microbiota	The sites with peri-implant mucositis presented with a less diverse and less anaerobic microbiome. Exposure to CHX, resulted in microbial changes after both 1 and 3 months.

## Data Availability

The datasets used and/or analyzed during the current study are available from the corresponding author on reasonable request.
